# The Classic: Walldius, Börje: “Arthroplasty of the Knee Using an Endoprosthesis: 8 Years’ Experience”, Acta Orthopaedica Scandinavica, 1960; 30: 1, 137–148

**DOI:** 10.3109/17453671003667192

**Published:** 2010-03-31

**Authors:** Olle Svensson

**Affiliations:** olle.svensson@orthop.umu.se

## Introduction

Börje Walldius was born in southern Sweden in 1913, but spent most of his professional life in Stockholm where he died in 1998. He published his ground-breaking thesis on knee prostheses at the Karolinska Institute in 1957.

At one staff meeting (personal communication), when discussing how to treat a rheumatic patient with severe bilateral knee destruction—when the only available surgical treatment was arthrodesis—Walldius suddenly took out a gadget from his pocket and said, “why not try this one”? It was a home-made methacrylate hinge prosthesis, designed by himself and manufactured by his brother, the owner of a toy factory. Carl Hirsh, later professor at the Karolinska, reacted with a stream of negative comments. However, Sten Friberg, Regius Professor and rector of the Karolinska, took the plastic gadget thoughtfully, carefully tested its range of motion, looked at it from all angles, and then walked absent-mindedly out of the room. A few minutes later, Friberg’s head nurse ran into the room and commanded: “Dr Walldius, go immediately to Professor Friberg’s office!”. Naturally, he expected to be sacked, but in the office Friberg, smiling and still playing with the prosthesis, said amicably, “OK, let’s do it tomorrow. Allow me to assist you”. The rest is a well-known success story.

Walldius, a hot-tempered and stubborn man, was certainly not afraid of expressing his personal opinions at meetings with great devotion, so great that he twice suffered heart attacks in action during academic disputes. On one occasion, the orthopods who were present (including your correspondent) managed to give him heart-lung resuscitation, so he revived and could be taken to the intensive care unit. Next day, he complained that his spectacles were broken.

Although a maverick, Göran Walldius is still one of the internationally best-known Swedish orthopedists ever, a pioneer and a free-thinker. One cannot help wondering what he would have said if he had had to convince a modern ethics committee about his first knee prosthesis. 

