# Supplementary material for: The Classic: Walldius, Börje: “Arthroplasty of the Knee Using an Endoprosthesis: 8 Years’ Experience”, Acta Orthopaedica Scandinavica, 1960; 30: 1, 137–148
Source: Acta Orthop. 2010 Mar 31;81(1):21–33. doi: 10.3109/17453671003667192 (PMC2856201; doi:10.3109/17453671003667192)
Supplement: Supplementary file 1 [file ORT-1745-3674-81-021_art.pdf]

FROM THE ORTHOPAEDIC DEPARTMENT OF KAROLINSKA INSTITUTET, STOCKHOLM (HEAD: PROFESSOR STEN FRIBERG)

ARTHROPLASTY OF THE KNEE USING AN ENDOPROSTHESIS

8 years' experience

By

BÖRJE WALLDIUS

In 1951 the author elaborated a method of operation for arthroplasty of the knee where, after resection of the joint, an endoprosthesis of acrylic resin, constructed as a hinge joint, was inserted. Primary results after this method were considered good.

Now, 8 years after the first operations were performed, a number of questions may with reason be considered.

Have the good primary results continued during this period?

Have late complications occurred since the introduction of this method, which may have suppressed or eeven reversed the optimism?

Has the operation any chance of becoming a "routine method" or is it only suitable for certain extreme cases?

Has the method proved to be suitable for the mechanical and biological requirements?

MATERIAL

The total number of operated patients is 51. 13 patients have been operated bilaterally. The total number of arthroplasties is therefore 64.

Indications for operation:

Rheumatoid arthritis	43 cases
Degenerative arthritis	5 cases
Morbus Bechterew	2 cases
Status post septic. arthritis	1 case

In order to be able to judge the results it is necessary to review the indications for operation. The first operations using a new method are

regarded as experimental. One is obliged to try and reduce the risk of impairment after operation to the minimum. This has been achieved by chosing those patients who are already so disabled that an unsuccessful operation could hardly cause a change for the worse. Those patients were mostly severely disabled rheumatics with flexion contractures and pains in the knee joints, where all conservative treatment had failed to help.

20 patients have been "wheel chair" cases. 10 of these have had bilateral arthroplasties performed. Of these 10 one had been in a wheel chair for 11 years, one for 10 years, one for 9 years, one for 7 years, one for 5 years, two for 3 years, and three for 2 years. The remaining patients had had great difficulty in using crutches or walking sticks, could manage a few steps indoors or only very short distances out of doors.

A significant atrophy of the quadriceps muscle had been present. 15 of the patients were, owing to the forced lack of movement, rather corpulent.

In many cases the suffering had caused mental depression. One patient was transferred from a psychiatric clinic, to which she had been admitted after an attempt to commit suicide.

The age of the operated patients varied between 29–79 years, with an average of 51 years.

The above-mentioned features have generally been regarded as contra-indications for arthroplasty of the knee joint. This must be considered when evaluating the results.

RESULTS

There were very good results with 41 arthroplasties (64 %), good results with 6 arthroplasties (10 %), poor results with 17 arthroplasties (26 %). When previous arthroplasty methods were used the results were about 50 % good and 50 % bad. The endoprosthesis method has, in spite of the most unfavourable conditions, shown an increase of good results by 24 %. A very good result means that the patient has a pain-free, stable joint with a range of mobility of 50°–90°. The average range of mobility in this group is 81°. Walking ability has been regained or greatly improved.

One of the patients with a good result has persistent pain, though not severe, and he walks without a limp. Two patients lack full stability in the joint. Two patients have become permanently bedridden as

a result of their progressive diseases, polyarthritis and Morbus Bechterew. The operated knees, however, have a mobility range of 80° and 90° respectively and are considered to be in good condition compared with the rest of the joints which have been completely destroyed. These patients are quite unable to walk or even stand upright. One patient has only 45° range of mobility but is otherwise fit.

Time of observation: for the whole material this covers a period of 8 years to 2 months. In 7 cases of successful arthroplasties the observation period was 8–5 years, in 13 cases 5–3 years, in 18 cases 3–1 year and in 9 cases less than 1 year.

Complications: Complications can be grouped into those causing a poor final result and those not influencing the result. Moreover, there are early complications occurring at the time of operation, and late complications.

Early complications consisted of pulmonary embolism in one case, and septicaemia in another, both with a fatal outcome.

In one case a disruptured wound, depending on insufficient immobilization, became infected and led to amputation. Another case with infection of the wound and paralysis of the peroneal nerve led to the same result.

In a case of Morbus Bechterew where the intention was to enable the patient to obtain a sitting position, the prosthesis was extracted after skin necrosis and infection.

In three cases considerable rigidity in the joint was present postoperatively. A possible explanation for this may be that owing to the acrylic prosthesis being a little too big, an electric drill was used to remove the protruding parts, after the prosthesis had been inserted. It was therefore impossible to avoid acrylic particles settling in the joint and causing irritation resulting in fibrosis of the joint. In order to relieve the rigidity, the patella was extirpated, but without success. An infection flared up and an arthrodesis was finally performed. This resulted in an ankylosis and healing of the infection.

In 4 more cases infection of the wound made it necessary to perform an arthrodesis, with ankylosis and healing of the infection. As regards walking ability these patients with arthrodesis have been improved by the operations.

Consequently the greatest risk involved by operation is infection. An infection in the skin has only a short distance to the joint. In a number of cases the sutures have been removed too soon and the wound has gaped open. Similarly physiotherapy has been commenced before

the wound has been properly healed. Complete healing usually takes 3 weeks.

A resistant strain of staphylococcus aureus has been a difficult antagonist.

A persisting rigidity with only 20° range of mobility + pain was registered in one case.

In two arthroplasties the femur part was driven in at a wrong angle, so hindering full correction of a flexion contracture.

Of the late-occurring complications there was one case of septic arthritis. The patient had had an open intraarticular fracture which was followed by septic arthritis and a fistula which greatly lengthened the time of healing. There were big adhesive scars on the anterior side of the joint and the knee had a varus position. This became more pronounced by a partial amputation of the foot, also in a varus position. It was suggested that the leg should be amputated at thigh level, but the patient preferred to undergo an arthroplasty of the knee first. After operation the joint had a mobility range of 35° but 14 months later the infection flared up again and a new septic arthritis made amputation necessary.

Fracture of the medullary pin of the prosthesis has necessitated the change of prosthesis in 2 cases. In one case the patient fell and injured the knee, 4 years and 7 months after the operation. In the second case it was a fatigue fracture. After change of the prosthesis good results were again achieved.

In one case of Morbus Bechterew where all the joints of the body were ankylosed a range of mobility of 20° was obtained in the knee. After operation the previous flexion contracture of 60° could be adjusted. This patient had also had a bilateral arthroplasty of the hip. After 11 years in a wheel chair she learnt to walk.

Among complications not causing impairment there were 2 cases with a lesser degree of paralysis of the peroneal nerve. This was repaired in both cases.

Necrosis of the skin in 2 cases and thrombosis in another did not cause any later inconvenience.

This applies also to 4 cases with fracture of the tibial pin of the prosthesis. This can be revealed roentgenologically but not clinically. The stability of the joint has been maintained due to the fact that the central part of the prosthesis is firmly fixed in the condyles. The greater part of its circumference is surrounded by an osteophytic wall.

Summarizing the complications it may be said that most of them

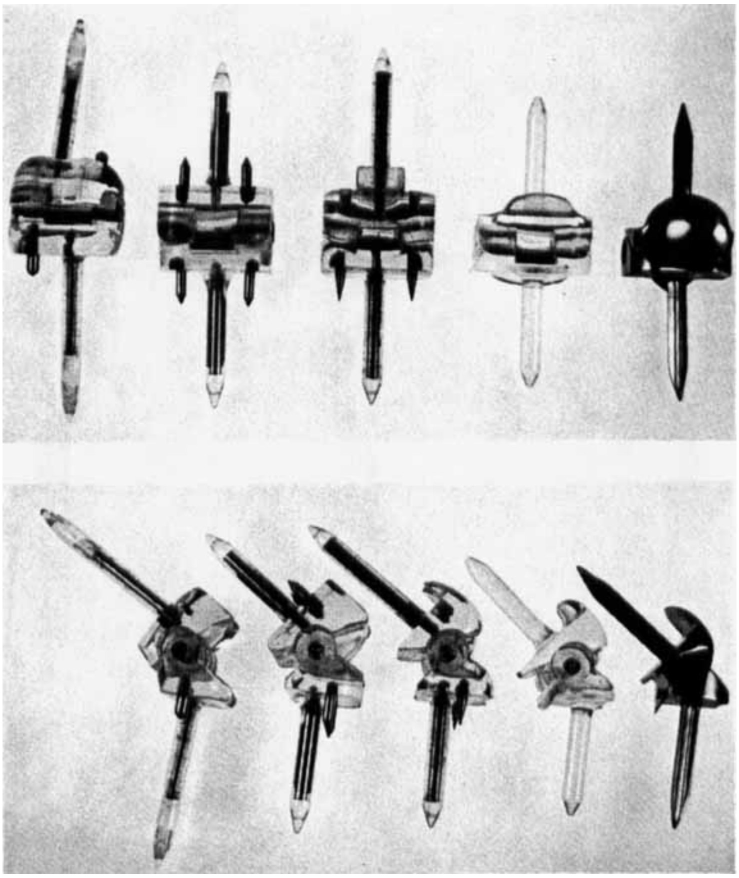

Fig. 1.

occur in connection with operation, and that late complications are relatively few. The greatest risk involved is of infection. This difficulty can be overcome by performing an arthrodesis, which has produced ankylosis in all the cases.

PROSTHESIS

The construction of the prosthesis has been modified (Fig. 1). At first the medullary pins were long and could cause conflict with the cortex, and now they have been shortened. In the first prosthesis there were also small pins of stainless steel on the central part to hinder rotation of the prosthesis around its longitudinal axis. These have now

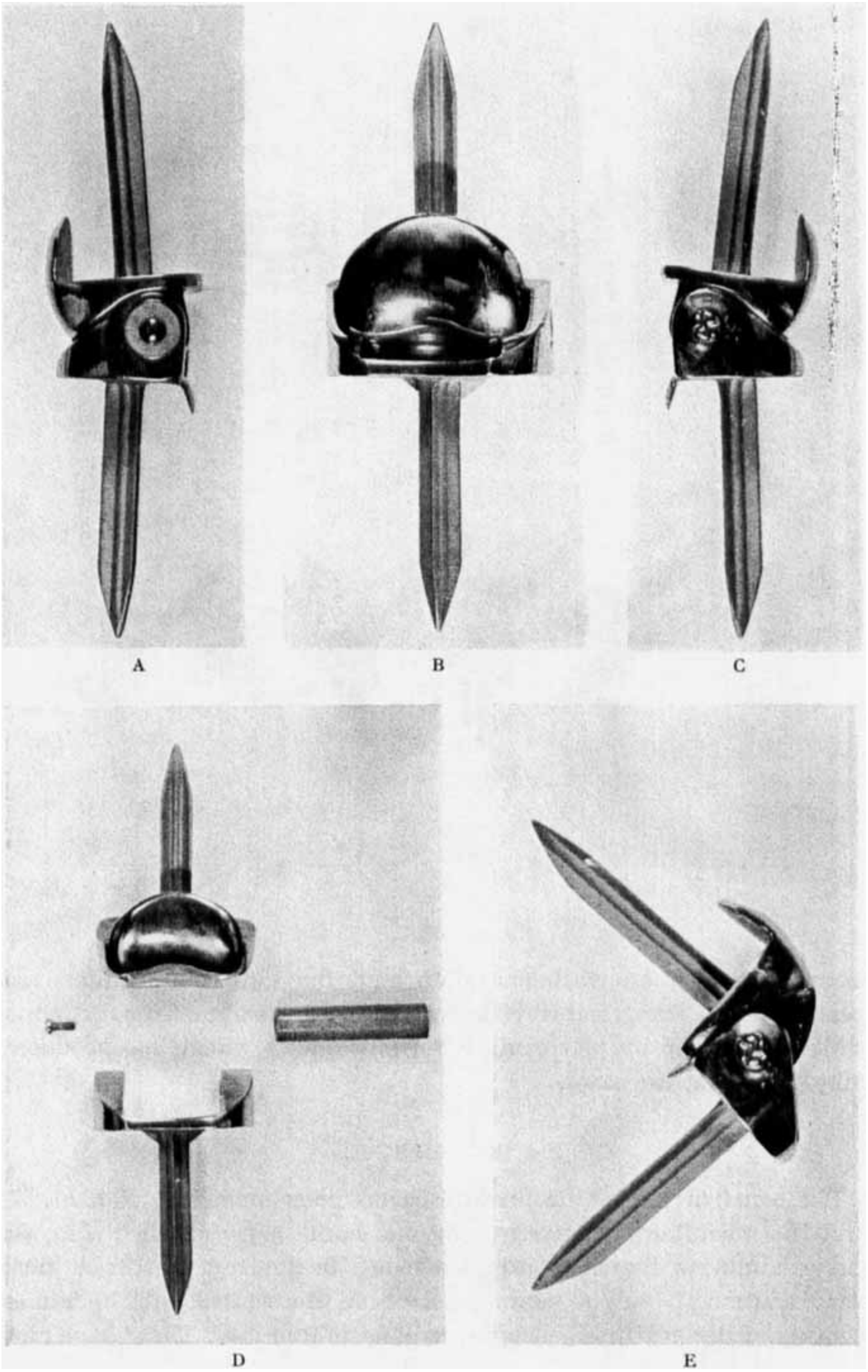

Fig. 2.

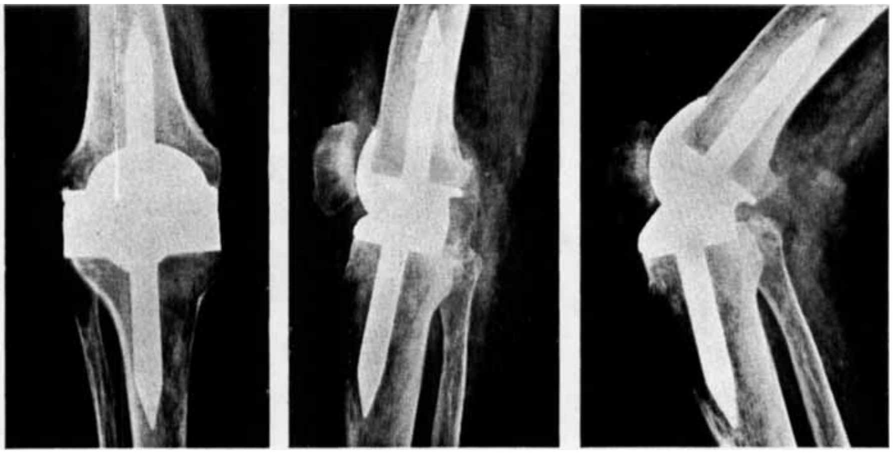

Fig. 3.
Roentgenograms with vitallium prosthesis.

been replaced by lips which produce the same effect, and moreover, the lip on the femur part forms a joint surface for the patella.

The first prostheses (58) were made of acrylic resin, now vitallium is used since it is found to be stronger and more durable (Figs. 2–3). The axis is covered with a layer of teflon, which has a low friction coefficient and great resistance to wear and tear. It was possible to manufacture the prosthesis in smaller sizes. The height of the central part was reduced from 48 mm. in the first prosthesis to 28 mm. in the vitallium prosthesis. The prosthesis is manufactured by AB. Stille-Werner in Stockholm.

OPERATION
(Figs. 4–15)

The operation should be porformed in a bloodless field. All the various types of skin incision were used and the incision according to Textor proved most suitable. The capsule and ligamentum patellae are dissected at the level of the skin incision. The skin is not loosened from the capsule. The joint surfaces are resected as in an arthrodesis. A flat piece of bone about 1 cm. thick is taken from the tibia and one about 2 cm. thick from the femur. The posterior parts of the femur condyles and the posterior edge of the tibia are chiselled off in order to prevent conflict between these parts with bending movements in the joint. No special instrument is required for the insertion of the prosthesis. It is easy to

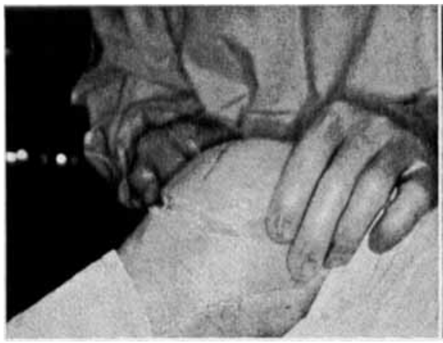

Fig. 4.

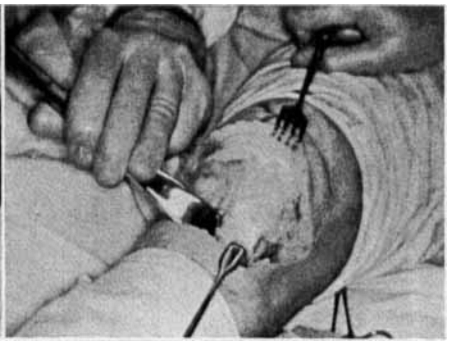

Fig. 5.

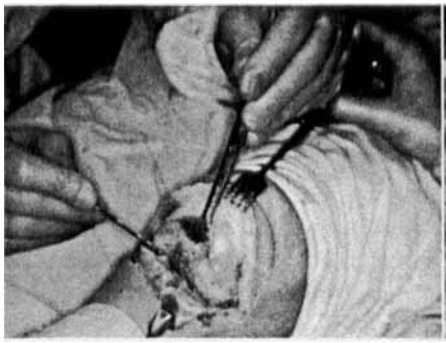

Fig. 6.

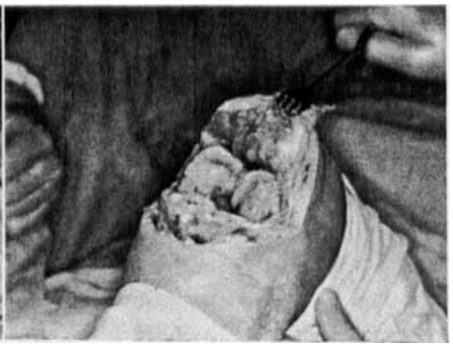

Fig. 7.

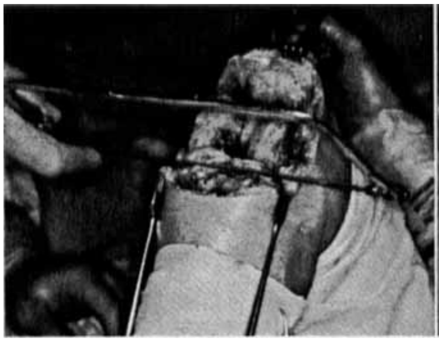

Fig. 8.

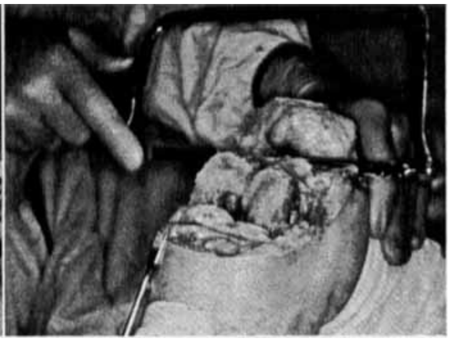

Fig. 9.

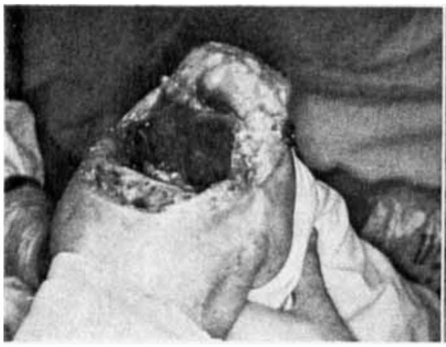

Fig. 10.

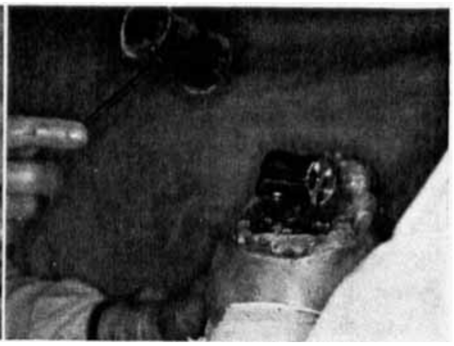

Fig. 11.

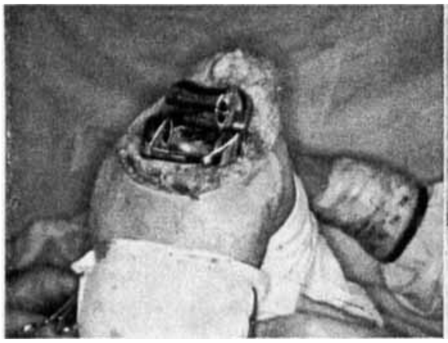

Fig. 12.

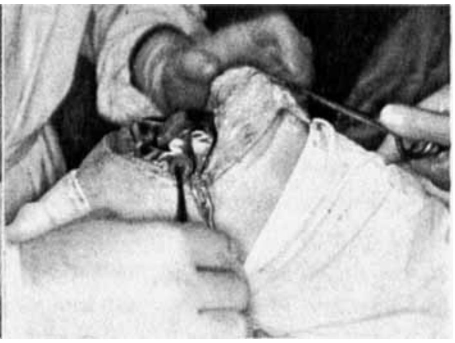

Fig. 13.

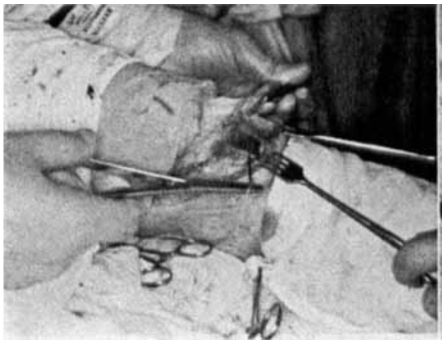

Fig. 14.

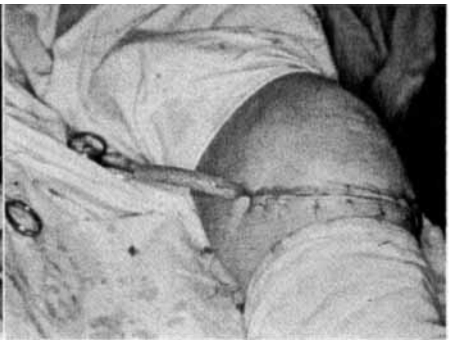

Fig. 15.

find the centre of the bone marrow cavity with a long probe. The two halves of the prosthesis are driven in so that a perfect fit is obtained between the resected surfaces and the surfaces of the centre part of the prosthesis. Adherences in the joint are excised. The patella is preserved and only bigger osteophytes are removed. Ligamentum patellae can usually be sutured double. The joint capsule is carefully sutured so that the prosthesis is completely covered. The edges of the capsule should therefore be well protected during operation in order to make suturing easier. Skin sutures. Wound dressing and dorsal plaster splint.

POSTOPERATIVE TREATMENT

The sutures and dorsal splint are removed, and exercise is started about 3 weeks after operation. When the patient is able to lift and straighten the leg, and the function of the quadriceps muscle is satisfactory, after another 3 weeks, then weight-bearing on the leg can be started.

BIO-MECHANICAL CONSIDERATIONS

It is interesting from the mechanical and biological point of view to compare the conditions for endoprosthesis operations in the hip and knee joints. In the hip joint the endoprosthesis is subject to a lever effect on weight-bearing and the prosthesis tends to slip from the bone. In the knee joint the weight-bearing is at a right angle to parallel, even surfaces without the same degree of lever effect as in the hip joint.

The surface of the bone supporting the prosthesis in the knee joint is about 2½ times larger than that in the hip joint. The weight per square unit in the hip joint becomes too great, resulting in atrophy of the collum femoris causing the prosthesis to slip. The bones in the knee joint, on the other hand, react by sclerosing. X-ray of the knee joint shows that the central part of the prosthesis becomes gradually surrounded by a bony formation. This is due partly to the sinking of the prosthesis a few mm. in the spongy tissue, at a relatively early stage, becoming surrounded by corticalis, and partly due to osteophytic formation. This increases the fixation of the prosthesis and stress on its medullary pin is decreased. The cone-shaped rarefaction of the bone structure sometimes occurring around the shaft of a Judétprosthesis, is found seldom, or not at all around the medullary pin in the knee prosthesis.

The hinge joint construction of the knee prosthesis garantees a full

congruency between the joint surfaces, a stable joint and a joint free from pain from the joint surfaces. It is therefore evident that the conditions for successful results of operations with endoprosthesis are greater in the case of a knee joint than in a hip joint.

CONCLUSION

This method has now passed the experimental stage. In spite of unfavourable conditions where indications are concerned, good results have been achieved and maintained during a long period of observation. Therefore, there seems to be grounds for gradually widening the indications for the use of this method. Even better results may be obtained in cases where conditions are more favourable.

SUMMARY

The author gives an account of 8 years' experiences with his method of arthroplasty of the knee using an endoprosthesis. 51 patients have been operated upon, 13 of these bilateral. That means 64 arthroplasties. The main indication has been rheumatoid arthritis followed by so extreme flexion-contracture in the knee that the walking ability during many years (max. 11 years) has been completely abolished or extremely restricted. The operative technique, which is simple, is described. Out of bio-mechanic point of view the conditions to reach good results by operations with an endoprosthesis in the knee joint seems to be better than in the hip joint. As it has been shown, that good primary results have been lasting during many years (max. 8 years), it seems to be justified successively to widen the indications for this operation.

RESUME

Un compte rendu de l'auteur des observations faites pendant une huitaine d'années, sur une méthode de l'arthroplastie du genou avec endoprothèse, élaborée par lui-même en 1951.

Le nombre des malades opérés remonte à 51, dont 13 ont été opérés aux deux genoux soit 64 opérations au total.

La principale indication opératoire a été arthrite reumatoide, accompagnée d'un défaut si grave d'extension du genou, supprimant ou restreingant fortement la faculté de marcher pendant plusieurs années (maximum 11 ans).

La technique opératoire, qui est extrêmement simple, est décrite. D'un point de vue biomécanique, les conditions pour obtenir de bons résultats des opérations par endoprothèse paraissent meilleures dans l'articulation du genou que dans celle de la hanche.

Il faut envisager d'amplifier successivement, les indication de cette opération, puisque cela s'est montré que les résultats primaires ont pu être maintenus pendant plusieurs années. (max. 8 ans).

ZUSAMMENFASSUNG

Der Verfasser gibt einen Bericht über achtjährige Erfahrungen mit seiner Methode der Arthroplastik des Kniegelenks, wobei eine Endoprothese verwendet wurde. 51 Patienten wurden auf diese Weise operiert, davon 13 auf beiden Seiten, zusammen ergo 64 Arthroplastiken. Die Hauptindikation stellte rheumatische Arthritis dar, die von so starken Beuge-Kontrakturen im Kniegelenk begleitet war, dass die Gehfähigkeit während vieler Jahre (maximal während 11 Jahren) vollkommen aufgehoben oder weitgehend eingeschränkt war. Die operative Technik, die einfach ist, wird beschrieben. Vom biochemischen Standpunkt ausgesehen scheinen die Verhältnisse zur Erreichung guter Resultate durch die Operationen mit Endoprothesen im Kniegelenk besser zu sein als in der Hüfte. Wie angedeutet wurde, hielten sich die guten primären Ergebnisse viele Jahre (maximal 8 Jahre) hindurch, sodass es gerechtfertigt erscheinen mag, allmählich die Indikation für diese Operation zu erweitern.

REFERENCE

Walldius, B.: Arthroplasty of the knee using an endoprosthesis. Acta Orthopaed. Scand. 1957. Suppl. 24.
